# Editorial Note: Design and implementation of a graphene–polyimide-based H-slot terahertz antenna for wireless and biomedical applications

**DOI:** 10.1371/journal.pone.0352489

**Published:** 2026-06-26

**Authors:** The PLOS One Editors

The *PLOS One* Editors issue this Editorial Note to inform readers that this article [1] includes terminology that differs from standards in the field. Specifically, Table 6 refers to “otherworldly effectiveness” instead of “spectral efficiency”.

The corresponding author stated that the terminology issue arose due to use of software for grammar and language correction during manuscript preparation.
